# The Promotion of Genomic Instability in Human Fibroblasts by Adenovirus 12 Early Region 1B 55K Protein in the Absence of Viral Infection

**DOI:** 10.3390/v13122444

**Published:** 2021-12-06

**Authors:** Tareq Abualfaraj, Nafiseh Chalabi Hagkarim, Robert Hollingworth, Laura Grange, Satpal Jhujh, Grant S. Stewart, Roger J. Grand

**Affiliations:** Institute for Cancer and Genomic Sciences, The Medical School, University of Birmingham, Birmingham B15 2TT, UK; TMA845@student.bham.ac.uk (T.A.); N.ChalabiHagkarim@bham.ac.uk (N.C.H.); r.hollingworth@bham.ac.uk (R.H.); LXG852@student.bham.ac.uk (L.G.); s.s.jhujh@bham.ac.uk (S.J.); g.s.stewart@bham.ac.uk (G.S.S.)

**Keywords:** adenovirus 12, E1B55K, DNA damage, DNA replication, DNA replication stress, DNA repair, DNA damage response

## Abstract

The adenovirus 12 early region 1B55K (Ad12E1B55K) protein has long been known to cause non-random damage to chromosomes 1 and 17 in human cells. These sites, referred to as Ad12 modification sites, have marked similarities to classic fragile sites. In the present report we have investigated the effects of Ad12E1B55K on the cellular DNA damage response and on DNA replication, considering our increased understanding of the pathways involved. We have compared human skin fibroblasts expressing Ad12E1B55K (55K^+^HSF), but no other viral proteins, with the parental cells. Appreciable chromosomal damage was observed in 55K^+^HSFs compared to parental cells. Similarly, an increased number of micronuclei was observed in 55K^+^HSFs, both in cycling cells and after DNA damage. We compared DNA replication in the two cell populations; 55K^+^HSFs showed increased fork stalling and a decrease in fork speed. When replication stress was introduced with hydroxyurea the percentage of stalled forks and replication speeds were broadly similar, but efficiency of fork restart was significantly reduced in 55K^+^HSFs. After DNA damage, appreciably more foci were formed in 55K^+^HSFs up to 48 h post treatment. In addition, phosphorylation of ATM substrates was greater in Ad12E1B55K-expressing cells following DNA damage. Following DNA damage, 55K^+^HSFs showed an inability to arrest in cell cycle, probably due to the association of Ad12E1B55K with p53. To confirm that Ad12E1B55K was targeting components of the double-strand break repair pathways, co-immunoprecipitation experiments were performed which showed an association of the viral protein with ATM, MRE11, NBS1, DNA-PK, BLM, TOPBP1 and p53, as well as with components of the replisome, MCM3, MCM7, ORC1, DNA polymerase δ, TICRR and cdc45, which may account for some of the observed effects on DNA replication. We conclude that Ad12E1B55K impacts the cellular DNA damage response pathways and the replisome at multiple points through protein–protein interactions, causing genomic instability.

## 1. Introduction

Human adenoviruses comprise a large family of approximately 90 different types divided into seven species (A to G). Normally they cause relatively mild infections of the respiratory and gastrointestinal tracts and the eye, depending on species. However, in immunocompromised patients, adenoviruses constitute a serious health risk, often resulting in high mortality rates [1,2]. In early studies it was shown that viruses from species A, such as adenovirus 12 (Ad12), were able to induce tumors in new-born rodents although viruses from species C (such as the very commonly studied Ad2 and Ad5) could not [3,4,5]. Injection of Ad12E1-transformed rodent cells into the syngeneic host could also produce tumors, whereas cells transformed with Ad5 or Ad2 DNA were only tumorigenic in athymic nude mice or immunosuppressed animals [6,7,8,9]. These observations allowed adenoviruses to be categorized as ‘DNA tumor viruses’. Consistent with this, Ad12 has also been shown to cause retinoblastoma-like tumors in baboons [10].

In slightly later studies it was shown that Ad12 could cause non-random damage to chromosomes 1 and 17 in human embryo kidney and human embryo lung cells [11,12,13]. These sites, referred to as Ad12 modification sites, have been mapped to chromosomes 17q21–22, 1p36, 1q21 and 1q42–43 and have marked similarities to classic fragile sites [11,13,14,15,16,17]. The four loci correspond to clusters of genes encoding small abundant structural RNAs. *RNU1* and *RNU2* loci contain tandemly repeated genes encoding U1 and U2 small nuclear RNAs (snRNAs), the *PSU1* locus is a cluster of degenerate U1 genes and the *RN5S* locus comprises tandemly repeated genes encoding 5S rRNA [16,18,19,20]. Adenovirus 5, on the other hand, appears to cause much more random breaks in human chromosomes [21,22]. Studies using Ad12 viruses with mutations in the E1 genes have demonstrated that expression of E1B55K is essential for chromosomal damage, whereas E1A and E1B19K had relatively little effect [23,24]. The random damage by Ad5, however, has been attributed to expression of E1A [21]. In view of these observations, we considered that further analysis of the ability of Ad12E1B55K to cause DNA damage and its effects on cellular DNA damage response (DDR) would be of considerable interest, particularly in view of our greatly enhanced understanding of DNA damage repair pathways since the original investigations were undertaken.

Expression of Ad12E1B55K is necessary for efficient viral replication, although this requirement appears to be less strict in the case of Ad5. Most functions of AdE1B55K, during viral infection, have previously been linked to its interaction with E4orf6 when it forms a ubiquitin E3 ligase in combination with cellular Cullins, Elongins and Ring box1, reviewed in [25,26,27,28]. The ubiquitylation of target E1B55K-binding proteins results in protein degradation in many but not all cases [25,26,27,28,29]. Thus, among the E1B55K-binding proteins already identified (reviewed in [27]), [29] a proportion are degraded while others remain intact [29].

Of the proteins degraded during Ad5 infection, an appreciable number of those studied in detail are involved in the DNA damage response (DDR), such as p53, MRE11, NBS1, BLM, DNA Ligase IV, TOPBP1, TIP60 and TAB182 [30,31,32,33,34,35,36]. Following adenovirus infection, there is an inactivation of the DDR which is recognized as a reduction in the activity of DDR kinases and, probably importantly from the virus’s point of view, a reduction in the ability of the cells to produce concatemers of viral genomes [32,37,38]. However, several reports have indicated that DDR proteins are localized to viral replication centers where it is believed that they contribute to replication, although their actual roles are as yet largely unknown. Similarly, DDR proteins have been identified at replication centers of other DNA viruses, such as HPV and KSHV, (reviewed in [39,40,41]).

Most previous studies of AdE1B55K have concentrated on its role during viral infection, when it is present with its partner, E4orf6, and functions as part of the E3 ligase. However, in the present study we were particularly interested in any unique functions of the protein linked to the DDR but distinct from its E3 ligase activity. Therefore, we have made use of human fibroblast cells expressing Ad12 E1B55K (55K^+^HSF). These cells are not transformed, nor are they immortal, but they do have an extended lifespan in culture [42]. Results have been compared to parental fibroblasts (HSFs) from the same donor. We have shown that Ad12E1B55K expression sensitizes cells to different damaging agents, seen as an increased number of DNA damage foci and increased activation of ATM, and, consistent with historical reports, increased genomic instability, seen as an increase in chromosomal damage and micronuclei occurrence. Furthermore, Ad12E1B55K has a detrimental effect on DNA replication, markedly increasing the number of stalled forks; it also increases the number of R-loops present. We suggest that most of these effects are probably due to the association of the viral protein with various DDR and replication complex proteins.

## 2. Materials and Methods

### 2.1. Cells and Drug Treatments

The derivation of HSFs and Ad12E1B55K-expressing HSFs (55K^+^HSFs) has been described previously [42]. Cells were grown in Dulbecco’s modified Eagle’s medium (DMEM) (Sigma-Aldrich), supplemented with 8% fetal calf serum (FCS) (Sigma-Aldrich, St. Louis, MO, USA). Adenovirus 12 Early Region 1 Human Embryo Retinal 2 (Ad12E1HER2) cells were also grown in DMEM supplemented with 8% FCS [43]. For various experiments, cells were treated with 1 µM camptothecin (CPT) (Cayman Chemicals, Ann Arbor, MI, USA), 2 mM hydroxyurea (HU) (Sigma-Aldrich), 3 µM aphidicolin (Sigma-Aldrich) and ionizing radiation (IR). In the CPT and aphidicolin experiments, the drug was left on the cells for the duration of the experiment, but treatment with HU was for 1 h only followed by removal of the drug and replacement with normal medium. Cells were also treated with 50 µM mirin or nocodazole (both from Sigma-Aldrich).

### 2.2. Western Blotting and Antibodies

Cells were grown to approximately 70% confluency and then harvested or treated with 2 mM HU, 1 µM CPT or ionizing radiation (4 Gy). Cells were washed in ice-cold phosphate buffered saline (PBS) and cell pellets were solubilized in 9 M urea, 40 mM Tris HCl, pH 7.4, 0.15 M β-mercaptoethanol. Cell lysates were fractionated by SDS-PAGE and proteins transferred to nitrocellulose membranes. Membranes were probed with antibodies against the following proteins: Ad12E1B55K (XPH9, raised against an Ad12E1B55K/β-galactosidase fusion protein, or a rabbit antibody raised against purified GST-Ad12E1B55K), p53 (DO1, a gift from David Lane), γH2AX (Millipore, Burlington, MA, USA), phospho-NBS1 (S343), phospho-KAP1 (S824), KAP1, SMC1, BLM, TOPBP1 and ATM (all from Bethyl Laboratories, Montgomery, TX, USA), phospho-CHK1 (S345) and phospho-CHK2 (T68) (both from Cell Signaling, Danvers, MA, USA), GAPDH, DNA-PK, PARP1, Ku80, CHK1, CHK2, NBS1, MRE11, MCM2, MCM3, MCM5, ORC1, ORC2, ORC3, ORC5, cdc45 and DNA Pol δ (all from Santa Cruz Biotechnology, Dallas, TX, USA), phospho-ATM (S1981) (R&D Systems, Minneapolis, MN, USA), H2B (Abcam, Cambridge, UK), TICCR (Sigma-Aldrich) and RPA32 (Calbiochem, San Diego, CA, USA). Antigens were visualized using ECL reagent (GE Healthcare, Chicago, IL, USA) and X-ray film (Kodak, Rochester, NY, USA).

Additional antibodies used in microscopical aspects of this study were raised against 53BP1 (Novus Biologicals, Littleton, CO, USA), Rad51 (Calbiochem), R-loops (S9.6, a gift from Angelo Agathanggelou, University of Birmingham, UK), Mitosin (Bethyl Laboratories, Montgomery, TX, USA) and Nucleolin (BD Bioscience, Franklin Lakes, NJ, USA), and BrdU (IdU, Becton Dickinson, Franklin Lakes, NJ, USA) and CldU (AbD SeroTec, Oxford, UK).

### 2.3. Co-Immunoprecipitation

Ad12E1HER2 cells were harvested in ice-cold PBS and lysed in 40 mM Tris HCl, pH 7.4, 1% NP40, 5 mM EDTA, 0.4 M NaCl. Insoluble material was removed by centrifugation at 100,000× *g* for 20 min. Lysates were incubated with appropriate antibodies overnight. Antigen–antibody complexes were isolated with protein G-agarose beads (Generon, Slough, UK) and fractionated by SDS-PAGE. Co-immunoprecipitating proteins were identified by western blotting.

### 2.4. Chromatin Preparation

Chromatin was prepared from 55K^+^HSFs and Ad12E1HER2 cells as described by Mendez and Stillman [44]. Briefly, cells were harvested in ice-cold PBS and resuspended in 0.34 M sucrose, 10% glycerol, 1.5 mM Mg_2_Cl, 10 mM KCl, 10 mM HEPES pH 7.9. Triton X100 was added to a concentration of 0.1% and cells were left on ice for 10 min. The cells were centrifuged for 5 min at 1300× *g* to give a nuclear pellet and a supernatant which was termed the ‘cytoplasmic fraction’ although it contained other organelles. After washing in the HEPES buffer, the nuclear pellet was resuspended in 3 mM EDTA, 0.5 mM EGTA, left on ice for 10 min and then centrifuged at 1700× *g* for 10 min. The pellet was taken as the chromatin fraction and the supernatant was the nucleoplasm.

### 2.5. Metaphase Spreads

Nocodazole at a final concentration of 200 ng/mL was added to sub-confluent HSFs and 55K^+^HSFs for 16 h. Cells were then harvested and subjected to hypotonic shock in 75 mM KCl for 30 min at 37 °C. Swollen cells were fixed with ethanol:acetic acid (3:1) solution and stored at −20 °C. Cells were dropped onto acetic acid-coated glass slides and allowed to dry overnight. Slides were then immersed in 5% Giemsa stain (Millipore) for 15 min followed by 5 min in water. The following day slides were mounted using Etellan mounting medium (Millipore) before metaphase abnormalities were scored using a Nikon Eclipse Ni microscope.

### 2.6. Immunofluorescence Microscopy

Cells were grown on glass cover slips for 24 h and then treated with 2 mM HU (for 1 h), 1 µM CPT or IR (2 Gy or 4 Gy). After the stated times, cells were fixed in 3.6% paraformaldehyde in PBS for 10 min and then permeabilized in 0.5%Triton X100 in PBS for 5 min before washing with PBS. Cells were stained with antibodies overnight, washed three times in PBS, and then stained with secondary antibodies for 1 h. DNA was stained with 4′,6-diamidino-2-phenylindole (DAPI). Fluorescence images were taken using a Nikon E600 Eclipse 333 microscope equipped with a 60× oil lens, and images were acquired using Volocity software 334 v4.1 (Improvision). Foci were counted in at least 100 cells in 3 separate experiments. Micronuclei were detected in cells fixed and permeabilized as described and stained for 5 min with DAPI. Micronuclei were also counted in at least 100 cells in 3 separate experiments.

### 2.7. Estimation of R-Loops

Cells were fixed and stained as described in the previous section. Cells were stained with the S9.6 antibody which recognizes R-loops and counterstained with an antibody against Nucleolin. Nuclei were stained with DAPI. The intensity of total nuclear staining due to S9.6 was measured and the intensity of the staining in the nucleoli subtracted as described [45]. Data were calculated using ImageJ.

### 2.8. DNA Fiber Analysis

DNA fiber analysis was performed essentially as described by Petermann and colleagues [46]. Briefly, cells were pulse-labelled with 25 μM CldU (Sigma-Aldrich) for 40 min, washed twice with medium and pulse-labelled with 250 μM IdU (Sigma-Aldrich) for 40 min. When cells were treated with 2 mM HU, it was included with the IdU. Labelled cells were harvested, and DNA fiber spreads were prepared as described [47]. CldU and IdU were detected by incubating acid-treated fibers with rat anti-BrdU antibody and mouse anti-BrdU antibody for 1 h. Slides were fixed with 3.6% PFA and incubated with Alexa Fluor 555 conjugated anti-rat IgG (Molecular Probes, Eugene, OR, USA) and Alexa Fluor 488-conjugated anti-mouse IgG secondary antibodies for 1.5 h. DNA fibers were viewed with a Nikon Eclipse E600 microscope and analyzed using ImageJ software. A summary of the protocol is shown in Supplementary Figure S1.

### 2.9. Statistical Analysis

The dot plot for the DNA fiber assay was performed using GraphPad Prism statistical software; (*n* = 3 independent experiments; >175 cells analyzed per repeat; Stats: unpaired *t*-test, * *p* < 0.05, ** *p* < 0.01, *** *p* < 0.001 and **** *p* < 0.0001). Quantification of cells with micronuclei classified in four categories: 1 micronucleus per cell, 2 micronuclei per cell, 3 micronuclei per cell and >4 micronuclei per cell (*n* = 3 independent experiments; >100 cells counted per repeat, mean ± SD, * *p* < 0.05, ** *p* < 0.01 and *** *p* < 0.001). Quantification of foci: *n* = 3 independent experiments; >100 cells counted per repeat, mean ± SD, * *p* < 0.05, ** *p* < 0.01 and *** *p* < 0.001. NS, not significant in all figures.

## 3. Results

**Figure 1:**
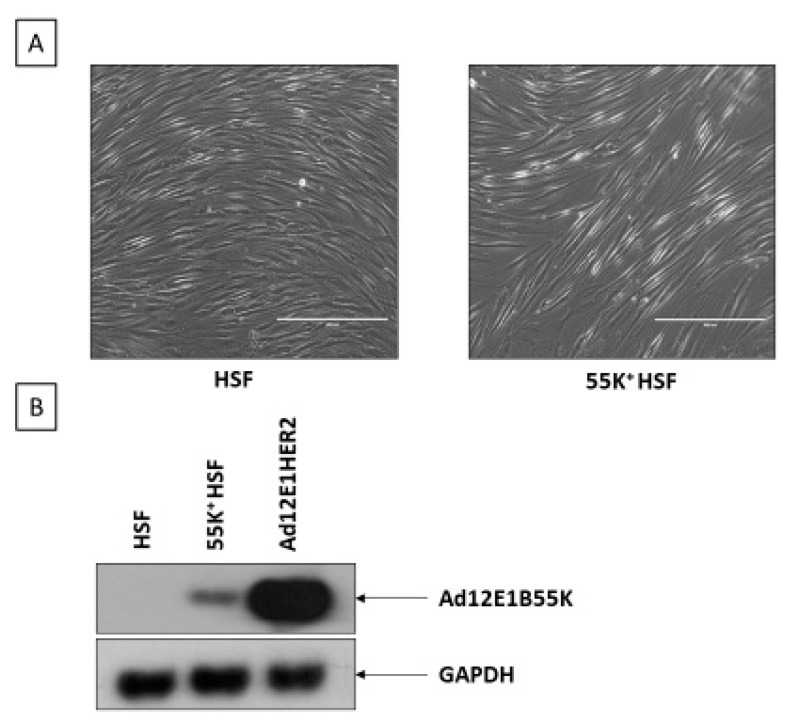
The morphology of HSFs expressing Ad12E1B55K (55K^+^HSFs). (**A**) Phase contrast micrograph of parental HSFs (left-hand panel) and 55K^+^HSFs (right-hand panel). The bar represents 400 µm. (**B**) Western blot of HSFs, 55K^+^HSFs and Ad12E1HER cells probed with antibodies against Ad12E1B55K and GAPDH.

To determine to what extent the AdE1B55K protein influences the cellular DNA damage response and DNA replication stress in the absence of E4orf6 and, therefore, the viral E3 ligase activity, we have made use of HSFs expressing Ad12E1B55K (55K^+^HSF) [42]. The cells have an appreciably extended life span but are not immortal or transformed and are similar in appearance to the parental cells (Figure 1A,B). It is notable that they express Ad12E1B55K at an appreciably lower level than Ad12E1 transformed human cells, as shown in Figure 1B.

### 3.1. Ad12E1B55K Induces Genomic Instability in Human Skin Fibroblasts

**Figure 2:**
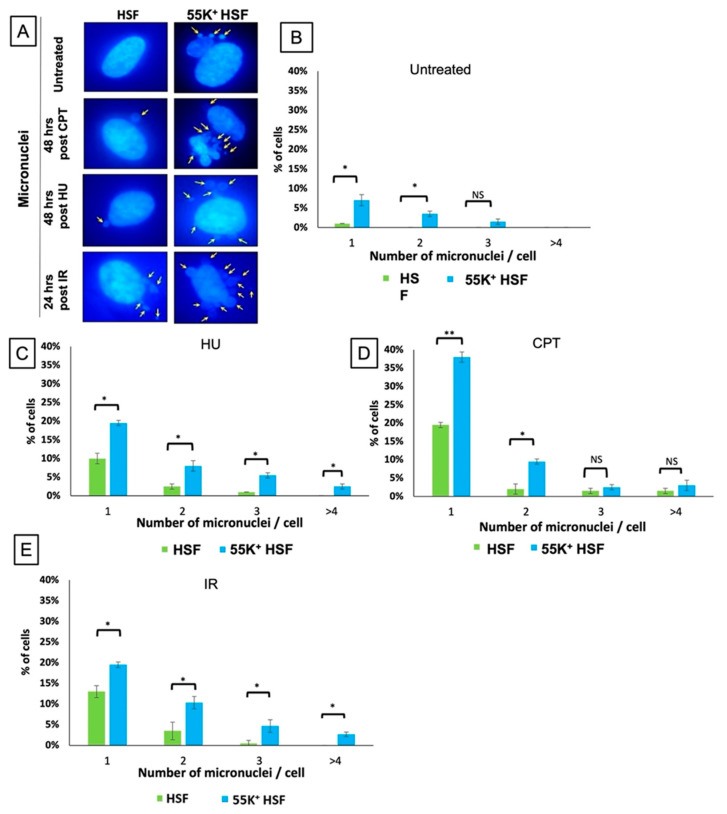
Micronuclei present in 55K^+^HSFs. HSFs and 55K^+^HSFs were exposed to DNA damaging agents, fixed after 48 or 24 h, and stained with DAPI. (**A**) Representative images of HSFs and 55K^+^HSFs (arrows indicate micronuclei). (**B**) Micronuclei observed in untreated cells. (**C**) Micronuclei observed 48 h after treatment with 2 mM HU. (**D**) Micronuclei observed 48 h after treatment with 1 µM CPT. (**E**) Micronuclei observed 24 h after treatment with IR (4 Gy). (100 cells were counted for each treatment: *n* = 3 independent experiments. Mean ± SD, * *p* < 0.05, ** *p* < 0.01 NS, not significant.

Previous studies have indicated that Ad12E1B55K induces chromosomal breaks at specific fragile sites and that these are similar to those induced by exposure to low doses of DNA-damaging agents [11,13,15,17,22,24]. However, these experiments were largely performed in the context of adenovirus 12 infection. To obtain a direct indication of the effect of the viral protein on genomic stability, the number of micronuclei was counted in normally dividing HSFs and in 55K^+^HSFs (Figure 2B). Micronuclei were also counted 24 or 48 h after treatment with HU, camptothecin or IR (Figure 2C–E). Micronuclei are rare in untreated HSFs but can be seen in 5–10% of the cells expressing Ad12E1B55K (Figure 2A,B). The micronuclei tended to be generally similar in size, although an occasional large one was visible, and clustered close to the DAPI-stained nucleus. In cells treated with DNA-damaging agents there are notably higher levels of micronuclei in 55K^+^HSFs, suggesting that Ad12E1B55K interferes with the cellular response to DNA replication stress and to DNA damage quite separately from its well-characterized ability to degrade DDR proteins with E4orf6 (Figure 2C–E).

**Figure 3:**
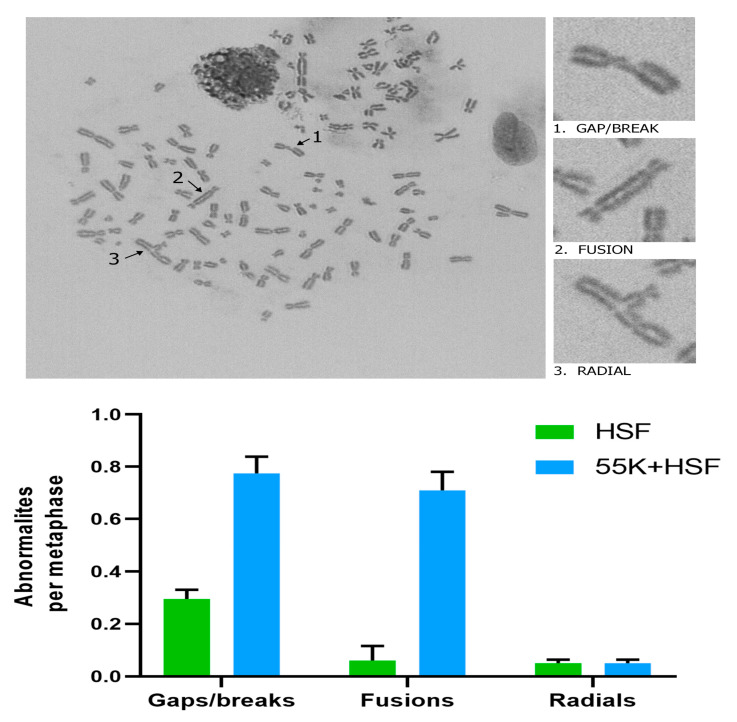
Metaphase spreads of 55K^+^HSFs. HSFs and 55K^+^HSFs were processed as described in the Materials and Methods section and the metaphase spreads prepared. A representative spread from 55K^+^HSFs is shown in the upper panel. Average numbers of abnormalities from 50 metaphase spreads from each cell population is shown in the histogram (*n* = 2).

In the original investigations of the effects of Ad12 on human chromosomes, gaps and breaks were observed in chromosomes 1 and 17 [11,12,13]. We were interested to see if there were more chromosomal abnormalities in the 55K^+^HSF cells. In the representative 55K^+^HSF metaphase spread shown in Figure 3, gaps, breaks and radials can be seen. Comparison of spreads from HSFs and 55K^+^HSFs showed appreciably more damage in cells expressing Ad12E1B55K (Figure 3). (No attempt was made to perform a more detailed analysis to see which chromosomes were particularly susceptible to damage.)

### 3.2. Ad12E1B55K Affects DNA Replication in HSFs and Leads to an Increase in R-Loop Formation

**Figure 4:**
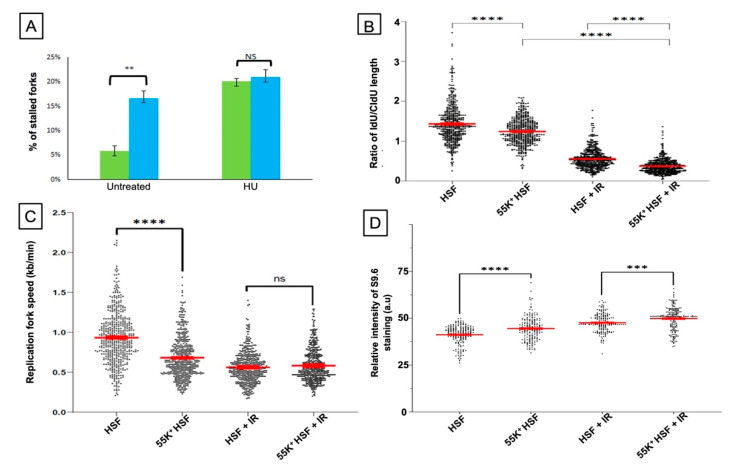
Expression of Ad12E1B55K in HSFs affects DNA replication and R-loop formation. (**A**–**C**), DNA fiber analysis was carried out on HSFs and 55K^+^HSFs in the presence or absence of 2mM HU, as described in Materials and Methods. (**A**) Percentage of HSFs and 55K^+^HSFs with stalled replication forks, before and after treatment with HU. (**B**) Ratio of Idu/Cldu tract length (taken as a measure of fork restart after fork stalling) before and after HU treatment. (**C**) Replication fork speeds of HSFs and 55K^+^HSFs, before and after treatment with HU. (**D**) Relative level of R-loops in HSFs and 55K^+^HSFs, before and after treatment with IR (4 Gy). Cells were fixed and stained with antibodies recognizing R-loops (S9.6) and Nucleolin and with DAPI. The intensity of R-loop staining in the nucleus, but outside the nucleoli, was calculated for cells in three separate experiments. For DNA fiber analysis, approximately 175 cells were analyzed for each repeat: *n* = 3 independent experiments. For the R-loop determination, 200 cells were measured for each repeat: *n* = 3 independent experiments. Unpaired *t*-test, ** *p* < 0.01, *** *p* < 0.001 and **** *p* < 0.0001). NS, not significant.

As treatment with the replication inhibitor, HU, has a marked effect on the genomic stability of 55K^+^HSFs (Figure 2C), we have investigated DNA replication in the two sets of cells. To examine DNA replication, use was made of DNA fiber assays [48] as outlined in the Materials and Methods section. Comparing 55K^+^HSFs with ‘normal’ HSFs, it is clear that expression of the viral protein increases the number of stalled replication forks observed, with a slight increase in new origin firing (Figure 4A). The efficiency of fork restart and replication fork speed is also decreased appreciably in 55K^+^HSFs (Figure 4B,C). To examine the effect of Ad12E1B55K on the response to DNA replication stress, cells were treated with 2 mM HU, which causes depletion of the nucleotide pool resulting in global replication fork stalling. In both populations, HU treatment resulted in a similar large increase in the number of stalled forks (Figure 4A), as expected, although the 55K^+^HSFs had a significantly reduced ability to restart replication efficiently (Figure 4B). The increase in stalled forks due to HU in the 55K^+^HSFs was only marginal. Examples of DNA fibers obtained in the study are shown in Supplementary Figure S1.

A common cause of DNA replication stress is the presence of DNA–RNA hybrid R-loops. To examine whether Ad12E1B55K affected R-loop formation, cells were stained with the S9.6 antibody [49] and the intensity of staining was calculated as described [45]. (R-loop staining in the nucleoli was subtracted from total nuclear staining.) There were significantly more R-loops, as indicated by the increased intensity of S9.6 staining, in the 55K^+^HSFs compared to the parental HSFs. In addition, staining with S9.6 was appreciably more intense, following IR, in cells expressing Ad12E1B55K (Figure 4D). Examples of R-loop staining are shown in Supplementary Figure S2.

### 3.3. Ad12E1B55K Protein Sensitises HSFs to DNA Damaging Agents

**Figure 5:**
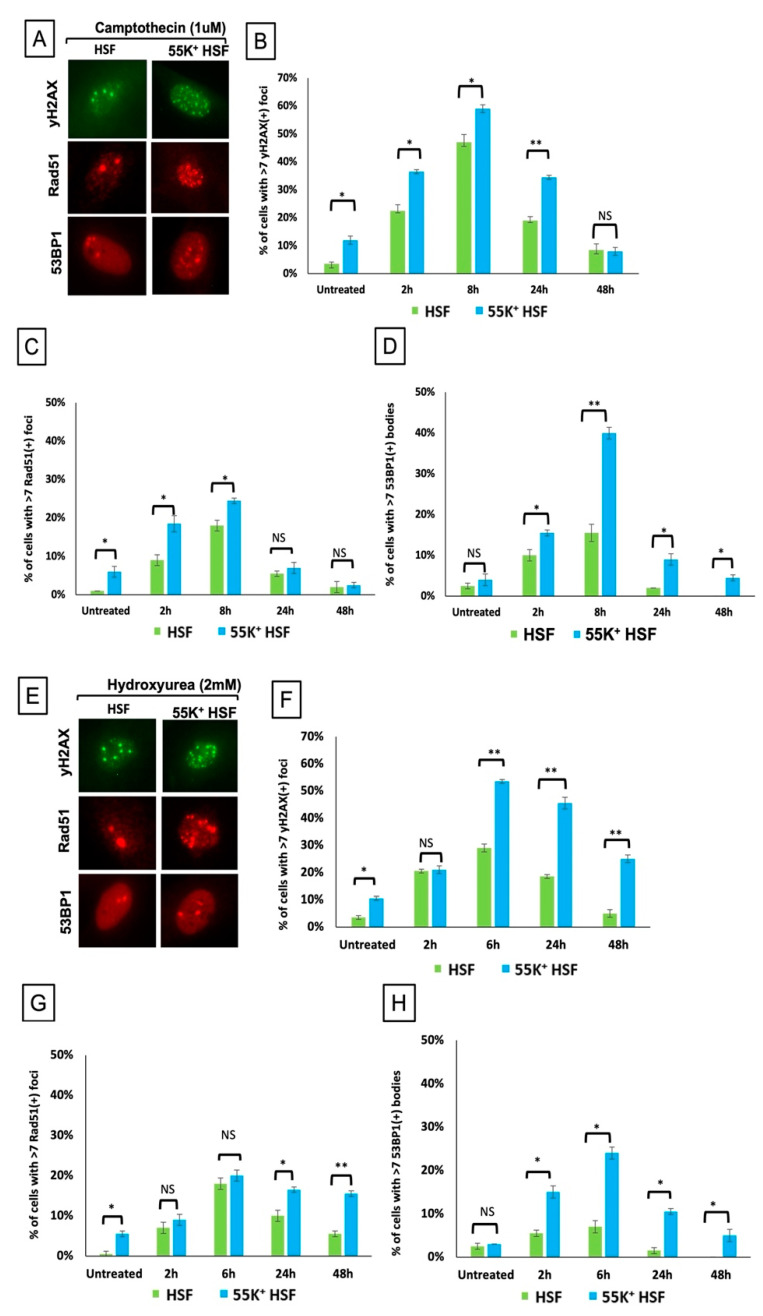

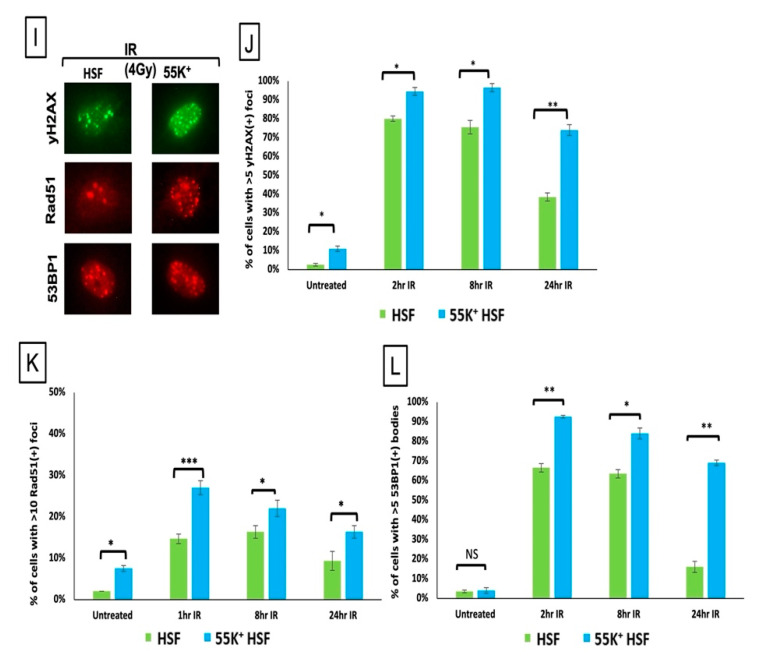
Expression of Ad12E1B55K in HSFs affects focus formation, following DNA damage. HSFs and 55K^+^HSFs were exposed to DNA-damaging agents and fixed at various times after treatment. Cells were stained with antibodies against Ad12E1B55K, γH2AX, 53BP1 and Rad51 and with DAPI. Panels (**A**,**E**,**I**) show representative images after exposure to CPT (1 µM), HU (2 mM) and IR (4 Gy), respectively. (**B**–**D**) cells exposed to CPT and stained for γH2AX (**B**), Rad51 (**C**) and 53BP1 (**D**). (**F**–**H**) Cells exposed to HU and stained for γH2AX (**F**), Rad51 (**G**) and 53BP1 (**H**). (**J**–**L**) Cells exposed to IR and stained for γH2AX (**J**), Rad51 (**K**) and 53BP1 (**L**). (*n* = 3 independent experiments; >100 cells counted per repeat; mean ± SD, * *p* < 0.05, ** *p* < 0.01 and *** *p* < 0.001). NS, not significant.

To understand better how Ad12E1B55K impinges on the DDR, we exposed 55K^+^HSFs and control cells to HU, camptothecin or IR and examined DNA repair foci formation over a 24- or 48-h time course, looking at recruitment of γH2AX, 53BP1 and Rad 51. In all cases, a greater number of foci were formed in the 55K^+^HSFs compared to the parental cells (Figure 5). For example, at 8 h after treatment, for each damaging agent, there are significantly more γH2AX, Rad51 and 53BP1 foci in the 55K^+^HSFs (Figure 5). This increased damage is consistent with the observed micronuclei, chromosomal aberrations, and increased replication fork stalling in cycling 55K^+^HSFs

**Figure 6:**
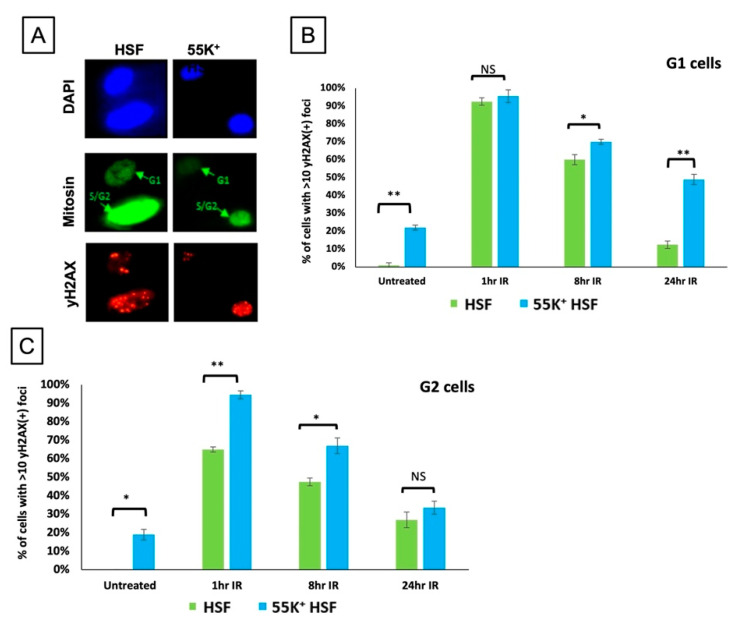
Ad12E1B55K affects DNA repair in HSFs in G2. Cells were treated with 3 µM aphidicolin for 45 min and then exposed to IR (2 Gy). Cells were fixed after the times shown and stained with antibodies against Mitosin and γH2AX and DAPI. γH2AX foci were counted in G1 (Mitosin negative) and G2 cells (Mitosin positive) (**A**) Representative images of Mitosin and γH2AX staining. (**B**) γH2AX foci in G1 cells. (**C**) γH2AX foci in G2 cells. (Approximately 100 cells were counted for each treatment at each time point for each repeat: *n* = 3 independent experiments; mean ± SD, * *p* < 0.05, ** *p* < 0.01). NS, not significant.

In a further experiment we have examined DNA damage focus formation in the different phases of the cell cycle. Cells were treated with 3 µM aphidicolin for 45 min, irradiated with 2 Gy and fixed after 1 h, 8 h or 24 h. Cells were stained with an antibody against Mitosin, which is expressed during the S, G2 and M phases of the cycle, and counter-stained for γH2AX (Figure 6), allowing the distinction between foci formed in G1 and those formed in G2 and S phases. In the Mitosin-positive cells it was also possible to distinguish between G2 cells (with a limited number of very distinct γH2AX foci) and S phase cells (with many more diffuse γH2AX foci). In the irradiated G1 cells (Mitosin negative), there are a similar number of foci in 55K^+^HSFs and HSFs at 1 h and 8 h post irradiation, although at 24 h there were appreciably more foci in the Ad12E1B55K-expressing cells. However, in the G2 cells there were appreciably more foci at 1 h and 8 h in the 55K^+^HSFs. We suggest that these data are indicative of deficiencies in the homologous recombination (HR) pathway, which is responsible for most DNA repair in G2, in cells expressing Ad12E1B55K.

We considered the possibility that Ad12E1B55K could localize to damaged foci following DNA damage. Generally, there was no co-localization between the viral protein and γH2AX and 53BP1 (Supplementary Figure S3). However, a few γH2AX foci appear to localize with more intense concentrations of Ad12E1B55K (marked with arrows in Supplementary Figure S3). It is not possible to say whether this is biologically significant at present. There appeared to be no co-localization with 53BP1.

**Figure 7:**
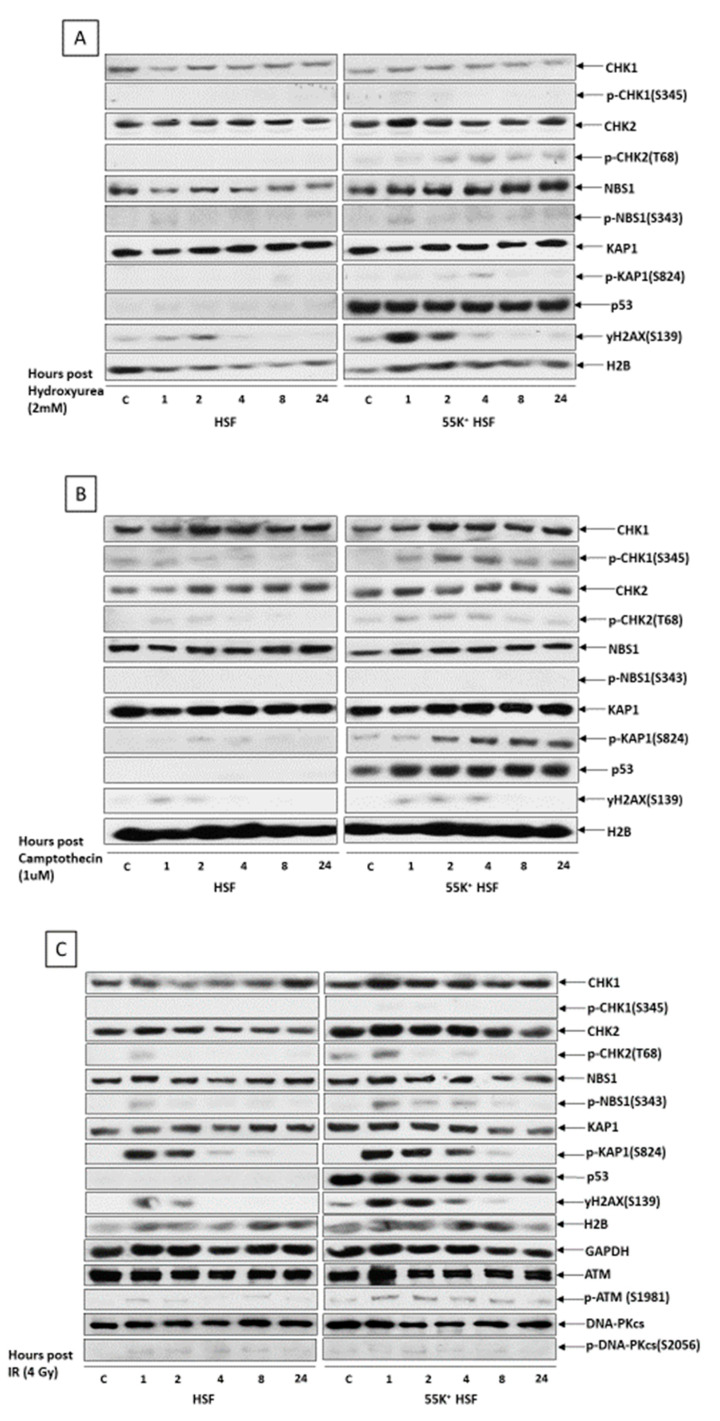
Ad12E1B55K sensitizes HSFs to DNA damaging agents. HSFs and 55K^+^HSFs were exposed to various DNA-damaging agents and harvested at the times shown. Cell lysates were fractionated by SDS-PAGE and western blotted using the antibodies shown. (**A**) HSFs and 55K^+^HSFs treated with 2 mM HU. (**B**) HSFs and 55K^+^HSFs treated with 1 µM CPT. (**C**) HSFs and 55K^+^HSFs treated with IR (4 Gy). c, untreated control cells.

In a further investigation, we exposed HSFs and 55K^+^HSFs to different DNA-damaging agents and examined the activation of the DDR over a 24-h time course, using western blotting coupled with antibodies recognizing known substrates of the PI-3-kinase-like kinases, ATM and ATR. The replication stress caused by HU will lead to single-strand breaks and ultimately to double-strand breaks in the DNA; similarly, camptothecin, a Topoisomerase 1 inhibitor, also causes double-strand breaks. It can be seen from Figure 7 that all three treatments produce a much more marked activation of the ATM pathway in the 55K^+^HSFs than in the parental cells, with CHK2, KAP1 and γH2AX being appreciably more highly phosphorylated (Figure 7). These data strongly suggest that Ad12E1B55K impinges on double-strand break repair pathways. This is consistent with previous studies, showing E1B55K/E4orf6-dependent degradation of the MRN complex, BLM, DNA Ligase IV and TIP60 in Ad5 infected cells [30,31,32,35]. Furthermore, some of these proteins are also degraded following Ad12 infection [50,51] (data not shown), with E1B55K probably acting as the binding component. Only very slight phosphorylation of DNAPK was observed (Figure 7C). Surprisingly, very limited phosphorylation of CHK1 was observed after HU treatment. It was expected that a much more marked response would be seen as the cells will be undergoing replication stress. The reasons for these observations are not clear at present. (In the blots shown in Figure 7, p53 is not obvious in the HSF cells; this is because relatively short exposures of the autoradiographs are shown so that those of 55K^+^HSFs are not too dark to be interpreted.) Interestingly, there does not appear to be an induction of p53 expression in the presence of Ad12E1B55K after DNA damage.

**Figure 8:**
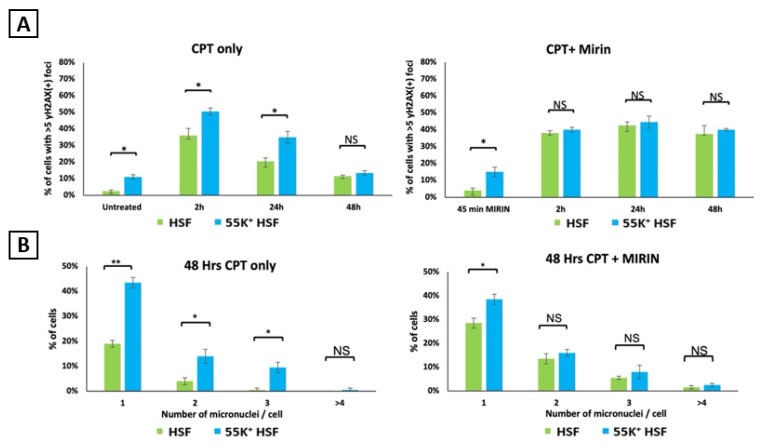
The effect of mirin of γH2AX foci and micronuclei formation in HSFs and 55K+HSFs. Cells were grown on glass coverslips for 24 h and treated with 50 µM mirin for 45 min. Cells were then treated with 1 µM camptothecin in the presence of mirin. (**A**) cells were fixed at the times shown and then stained for γH2AX. Foci were counted. (**B**) DAPI-staining micronuclei were counted after 48 h (*n* = 3 independent experiments; >100 cells counted per repeat; mean ± SD, * *p* < 0.05, ** *p* < 0.01). NS, not significant.

It is clear from the data presented in Figure 5 and Figure 7 that Ad12E1B55K has a marked effect on the DDR, probably compromising double-strand break repair and possibly other pathways. To investigate this further, we examined whether inactivation of MRE11 by the inhibitor mirin would duplicate some of the effects of Ad12E1B55K in the parental HSFs. 50 µM mirin was added to HSFs and 55K^+^HSFs for 45 min before treatment with 1 µM camptothecin. Cells were fixed after 2, 8, 24 and 48 h and γH2AX foci were counted (Figure 8A). The number of foci in 55K^+^HSFs was not appreciably affected by mirin at the earlier time points, although it was markedly increased at 48 h. However, there were significantly more foci in HSFs. Notably, at 24 h the number of γH2AX foci were very similar in the two populations in the presence of mirin, but in its absence, there were 1.7 times as many foci in 55K+HSFs. We suggest, based on these data, that Ad12E1B55K influences the cellular DDR, at least in part, by inactivation of the MRN complex. In addition, the number of micronuclei observed 48 h after damage was much more similar in the two populations in the presence of mirin than in its absence (Figure 8B). Thus, there were approximately four times as many cells with two micronuclei per cell in the 55K^+^HSFs than in the parental cells in the absence of mirin but a comparable number in each in its presence. Virtually no HSFs were seen with more than three micronuclei in HSFs in the absence of mirin but there were similar numbers in the two populations when MRE11 was inhibited.

### 3.4. Ad12E1B55K Affects the Ability of HSFs to Undergo Cell Cycle Arrest following DNA Damage

**Figure 9:**
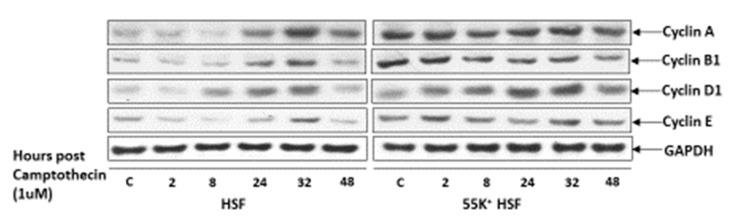
Cyclin expression in 55K^+^HSFs and HSFs following DNA damage. HSFs and 55K^+^HSFs were treated with 1 µM camptothecin. After 1 h the drug was washed out and the cells incubated in normal medium. Cells were harvested at the times shown and lysates subjected to western blotting using the antibodies shown.

In view of the widespread effects of Ad12E1B55K on the DDR and on DNA replication, we examined whether there were differences between both sets of cells in their ability to arrest their cell cycles following DNA damage. HSFs and 55K^+^HSFs were treated with 1 µM camptothecin and harvested at times up to 48 h. Cell lysates were western blotted for cyclins to assess progress through the cell cycle (Figure 9). In the HSFs there appears to be cell cycle arrest soon after DNA damage with a low level of expression of cyclins A, B1 and D1. After 8 h, cyclin D1 expression increases, presumably as cells re-enter cycle. After 24 h, there is an increase in cyclins A and B1 as cells go through S and G2 phases. On the other hand, in the 55K^+^HSFs the expression of cyclins remains high throughout the time course, suggesting that the cells do not undergo arrest, most probably due to inactivation of p53 (even though the expression is much higher than in the parental cells (Supplementary Figure S4)).

### 3.5. Association of Ad12E1B55K with Replication Machinery and DDR Pathway Components

To examine whether Ad12E1B55K influences DNA replication and the DNA repair pathways by affecting levels of relevant protein expression, samples of the 55K^+^HSFs and parental HSFs were western blotted using a variety of antibodies, as shown in Supplementary Figure S4. There were some differences in the expression of proteins associated with replication, such as reduced levels of MCM2, ORC1, ORC2 and ORC6 in 55K^+^HSFs (Supplementary Figure S4). There is a large overexpression of p53 in the Ad12E1B55K-expressing cells, as has been reported previously; this protein is transcriptionally inactive, presumably due to its association with Ad12E1B55K [52]. There are limited differences in expression for the other DDR proteins examined between the two cell types; for example, BLM is present at an increased level in 55K^+^HSFs (Supplementary Figure S4).

**Figure 10:**
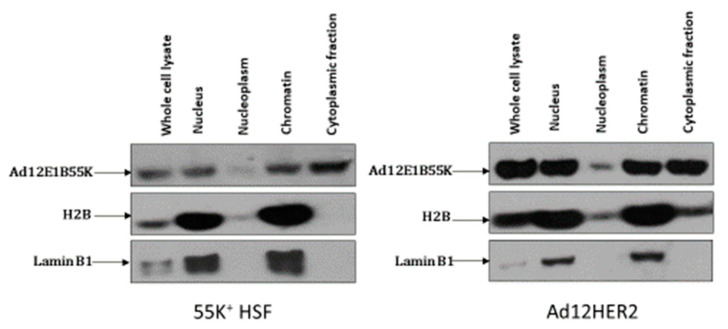
Ad12E1B55K localizes to chromatin in 55K^+^HSFs and Ad12E1HER2 cells. Cells were harvested and processed as described in the Materials and Methods. Fractions were western blotted using the antibodies shown. The cytoplasmic fraction also includes some organelles.

It is clear from the micrographs shown in Supplementary Figure S3 that Ad12E1B55K is predominantly nuclear. Therefore, initially we were interested to see if a proportion of the protein was associated with chromatin. Figure 10 shows that the nuclear fraction of the viral protein is present in the chromatin and not the nucleoplasm. A significant proportion is also present in the cytoplasmic fraction (Figure 10).

During Ad-mediated protein degradation, it has been shown or, in some cases, assumed that the E1B55K protein acts as the binding component for the cellular target to be ubiquitylated by the E3 Ligase [25,27,28,29]. It is possible, therefore, that the effects of Ad12E1B55K on DNA replication and on the DDR in the 55K^+^HSFs are due to its protein–protein interactions.

**Figure 11:**
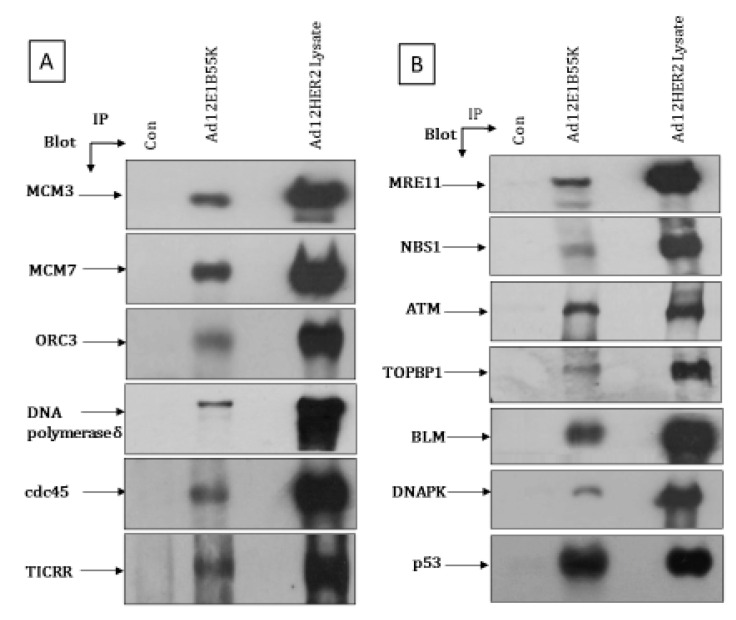
Co-immunoprecipitation of Ad12E1B55K with: (**A**), pre-replication complex and (**B**), DNA damage-response proteins. Ad12E1HER2 cell lysates (500 µg) were incubated with antibodies against Ad12E1B55K and collagen IV (non-specific binding control). Immunocomplexes were isolated with protein G-agarose beads and subsequently resolved by SDS-PAGE and western blotting, using antibodies against the proteins shown. Con: immunoprecipitation with collagen IV antibody.

In a more detailed analysis, we have carried out a limited series of co-immunoprecipitations, looking at whether Ad12E1B55K associates with various likely replication machinery and DDR pathway components (Figure 11). For this we have used Ad12E1HER2 cells as they can be grown more easily in much greater numbers than the fibroblast line and express E1B55K at a higher level (Figure 1). In Figure 11A, it can be seen that immunoprecipitation of Ad12E1B55K results in co-precipitation of MCM3, MCM7 and ORC3, indicating association between the viral protein and at least some members of the ORC and MCM complexes. No association was seen with ORC2, ORC6, MCM2 or MCM4 (data not shown). In addition, Ad12E1B55K binds strongly to DNA polymerase δ, TICRR (also known as Treslin and SLD3) and cdc45 (Figure 11A)—well characterized components of the replisome. No interaction with PCNA could be observed (data not shown). It is possible that binding to the MCM complex, ORC1 and/or DNA Pol δ could be responsible for some of the aberrations observed in DNA replication. Ad12EB55K also associates with p53, as has been observed previously, and MRE11 and NBS1, as might be expected based on the observation that they are targeted for degradation during Ad12 infection [50,51,52]. Co-immunoprecipitation of Ad12E1B55K with ATM, DNA-PK catalytic subunit, BLM and TOPBP1 was also observed (Figure 11B). It is likely that these interactions could have profound effects on the DDR.

**Figure 12:**
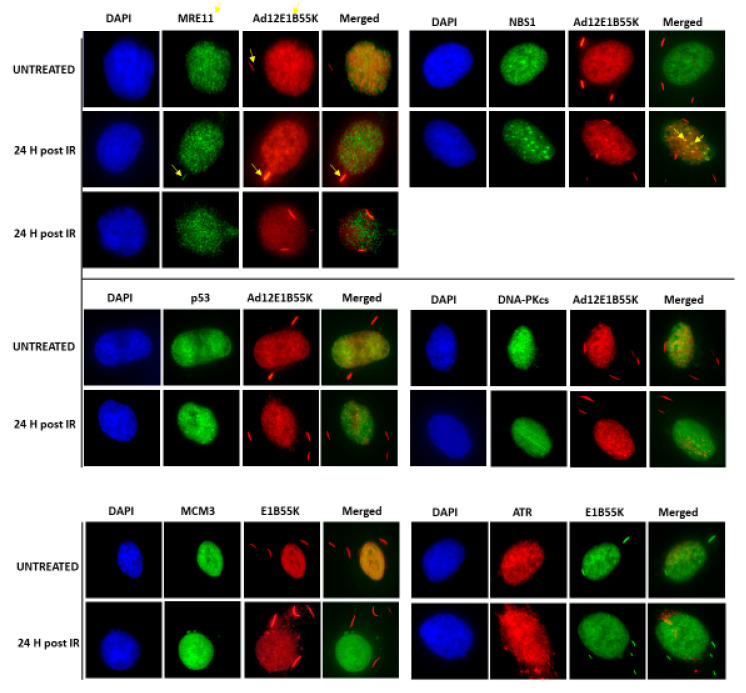
Co-localization of Ad12E1B55K with binding partners after DNA damage. 55K^+^HSFs were grown on glass coverslips for 24 h. Cells were treated or mock-treated with IR (3 Gy) and then fixed after 24 h. Cells were stained with the antibodies shown. In the upper left-hand set of images, arrows indicate Ad12E1B55K (red) or MRE11 (green) localizing to cytoplasmic tracks. In the upper right-hand set of images, arrows indicate tentative co-localization of NBS1 and Ad12E1B55K at DNA repair foci.

In the micrographs shown in Supplementary Figure S3 and in the original report [42], it is notable that a proportion of the Ad12E1B55K protein is present as very brightly staining cytoplasmic ‘tracks’ which appear not to be affected by DNA damage. We considered the possibility that these could be sites of interaction with some of the binding proteins identified in Figure 11. Co-staining for some of these, however, showed that only MRE11 had limited co-localization (marked with arrows in the left-hand upper section of Figure 12). We could not detect any of the other binding partners in the ‘tracks’, including NBS1. However, it did appear that there was some co-localization of Ad12E1B55K with NBS1 foci formed after IR treatment (marked with arrows in the right-hand upper section of Figure 12).

## 4. Discussion

It is now well-established that Group A and Group C adenoviruses target multiple key components of the DDR during viral infection (reviewed in [25,27,28]). This has been shown, in most cases, to be achieved through ubiquitylation and proteasome-mediated degradation, initiated by a complex between E1B55K, E4orf6 and cellular Cullins, Elongins and Rbx1. However, we considered the possibility that the E1B55K protein could impact DDR pathways in the absence of E4orf6. This was based on the historic observations that Ad12E1B55K was responsible for non-random damage to chromosomes 1 and 17 at low viral doses and in the absence of other viral proteins [11,13,16,20,24,53,54]. A synergistic effect between Ad12 and a DNA-damaging agent in the induction of these breaks has also been demonstrated [22]. Importantly, expression of the protein is essential for Ad12-mediated transformation of human cells in culture where it is possible that inactivation of the DDR could play a role [43,55]. To examine the relationship between the viral protein and the DDR, we have made use of HSFs expressing Ad12E1B55K (55K^+^HSFs) and compared results with the parental cells [42].

The expression of the Ad12E1B55K protein induces genomic instability in the HSFs, as shown by an appreciable number of micronuclei in growing cells, as well as an enhanced number of micronuclei in cells treated with DNA-damaging agents (Figure 2). Increased numbers of damaged chromosomes were also observed in metaphase spreads prepared from 55K^+^HSFs (Figure 3), consistent with the original reports [11,12,13,14,15]. As HU increases the number of micronuclei in 55K^+^HSFs to a much greater extent than in the parental cells, we considered it possible that Ad12E1B55K could cause DNA replication stress. From the results presented in Figure 4, it is clear that replication is compromised in the presence of the viral protein in unperturbed cells with a marked increase in stalled forks, reduced efficiency of replication fork restart and a decrease in replication fork speed. Following HU treatment, both populations of cells had a similar number of stalled forks and similar replication fork speed. However, fork restart was significantly more efficient in the parental cells than in 55K^+^HSFs. We suggest that the Ad12E1B55K protein has a major impact on DNA replication in the generation of stalled forks in addition to compromising efficient replication fork restart after stress. All these effects can contribute to genomic instability (reviewed in, for example, [56,57]). It seems likely that the interactions observed between Ad12E1B55K and components of the replisome (ORC1, MCM3, MCM7, DNA polymerase δ and cdc45) contribute to these effects (Figure 11).

During viral infection, adenoviruses target multiple aspects of DSB repair, with degradation and/or translocation of MRN components, BLM, DNA Ligase IV and TIP60 [30,31,32,35,58]. To examine whether the Ad12E1B55K protein in isolation would also affect the cellular response to DSBs, cells were exposed to different damaging agents and repair focus formation and phosphorylation of PI3K-related kinase substrates was monitored. Increased phosphorylation of ATM substrates in 55K^+^HSFs was seen, suggesting problems with the repair of DNA damage and perhaps the presence of additional damage due to checkpoint problems (see below) (Figure 7 and Figure 9). Similarly, an increased number of foci staining positive for γH2AX, Rad51 and 53BP1 were observed in 55K^+^HSFs, compared to controls at all time points after damage, again suggesting possible inefficiencies in DDR pathways and/or additional damage due to the effects of Ad12E1B55K (Figure 5). Ideally, a more detailed analysis of repair and resolution of DNA damage would have been undertaken to determine the relative contributions of increased damage compared to problems with repair. Interestingly, even in untreated cells an appreciable number of repair foci were visible, consistent with the proposition that Ad12E1B55K contributes to genomic instability in the absence of extraneous DNA-damaging agents (Figure 2 and Figure 3).

To determine which repair pathways were likely to be targeted, we examined γH2AX focus formation (in response to a low dose of IR) throughout the cell cycle by adding aphidicolin and delineating G1 and G2 cells by staining for Mitosin (Figure 6). Focus formation was similar for both populations of cells in G1 up to 8 h, where repair would be by NHEJ, whereas there were many more foci in the 55K^+^HSF G2 cells, when repair is predominantly by homologous recombination. The observation that there was a greater disparity at 24 h between the control and 55K^+^HSFs in G1 cells (compared to those in G2) is probably due to a breakdown in the aphidicolin cell cycle block (Figure 6). At 8 h, however, there are many more foci in the G2 cells when the cell cycle block is likely to be in place.

Examination of the cell cycle after DNA damage in 55K^+^HSFs suggests that the checkpoints are compromised (Figure 9). This is consistent with inactivation of p53 through direct interaction with Ad12E1B55K [50,52,59]. In the absence of transcriptionally active p53 the G1/S and G2/M checkpoints will not be functional. In the absence of one or more checkpoints, it is likely that more damage to the DNA will be incurred for a given treatment, giving rise to more damage foci and probably more activation of ATM (Figure 5 and Figure 7).

To investigate the effects of Ad12E1B55K further, we have examined the level of expression of several DDR proteins to rule out the possibility that differences seen are due to protein degradation, as it has previously been noted that loss of Daxx during Ad5 infection requires only E1B55K with no contribution from E4orf6 [60]. As Ad12E1B55K also appears to affect DNA replication, the level of expression of some components of the origin recognition complex (ORC) and the pre-replicative complex were examined. Some differences were observed in the expression of ORC proteins and in MCM2, possibly affecting DNA replication. There were some disparities in the expression of some DDR proteins between the two cell populations, such as BLM. In addition, p53 is massively overexpressed in 55K^+^HSFs, as has been reported previously, although this is likely to be transcriptionally inactive as already discussed [52,59]. We considered that the variations seen in the DDR proteins were probably not sufficient to be responsible for the marked difference in properties observed in the two cell populations, although this would need to be confirmed by further investigation. Moreover, the effects of high levels of transcriptionally inactive p53 are not clear [52].

To pinpoint possible targets of Ad12E1B55K more closely, a series of co-immunoprecipitations was undertaken. It is likely that the observed interaction of Ad12E1B55K with MCM3, MCM7, ORC3, DNA polymerase δ and cdc45 could affect ‘normal’ DNA replication and following replication stress. No association with other MCM or ORC proteins or PCNA was observed. In an examination of a limited number of the DNA repair proteins, Ad12E1B55K associated with the MRN complex components, MRE11 and NBS1. This would be expected, as MRE11 is degraded during Ad12 infection [50,51]. Co-immunoprecipitation of p53 has been described previously [52]. Similarly, association between Ad5E1B55K and BLM has been reported [30]. Interactions between AdE1B55K proteins and ATM and DNA-PK, however, represent novel targets which are likely to contribute to the DDR effects reported here. Whether comparable interactions occur with E1B55K proteins from other adenovirus serotypes, such as Ad5, is unknown at present. It seems likely, however, that these associations will be of importance during viral infection.

We have also examined the sub-cellular distribution of E1B55K in the 55K^+^HSFs. Whilst much of the protein is nuclear and associated with chromatin, an appreciable amount is present as ‘tracks’ within the cytoplasm (Figure 12 and Supplementary Figure S3B). Previous studies, particularly of Ad5, have reported the presence of E1B55K in large cytoplasmic juxtanuclear aggresomes in Ad5E1-transformed cells [61,62,63]. p53 and the MRN complex are also present. During Ad5 infection, similar aggresomes occur, induced by Ad5E4orf3 and/or orf6 and E1B55K [62,63]. These are considered to be the sites of MRN degradation [62,63]. From their location and appearance, we consider it unlikely that the ‘tracks’ seen in 55K+HSFs are aggresomes. Furthermore, we could see no co-localization of p53 at these structures (Figure 12). At present, we have no further knowledge of their origin.

The effects of E1B55K on host cell pathways described here may not be very relevant to Ad12 infection since, in that case, E1B55K will be accompanied by E4orf6 and the target proteins will probably be ubiquitylated. Whether this would result in proteasome-mediated degradation remains to be established [29]. However, it is likely that disruption of DNA replication, an increase in R-loop formation and interference with the DNA damage response will all favor AdE1-mediated cellular transformation and tumor formation. It is well known that the interaction of both Ad2/Ad5 and Ad12 E1B55K with p53 inhibits its transcriptional transactivating properties, which is considered to be important for efficient transformation [64]. However, it has also been demonstrated that properties of E1B55K, other than association with p53, contribute to transformation of rodent cells by AdE1 [64,65,66,67]. It was suggested that an interaction with the MRN complex, as well as an ability to form multi-protein complexes, could be factors favoring the transformation process [66]. In that study it was not shown whether the mutant E1B55K proteins induced any of the effects on DNA replication or the DDR reported here.

Despite the multiple effects of AdE1B55K on host cells, it cannot transform mammalian cells in isolation; AdE1A is essential for the process [68,69]. However, expression of AdE1A alone results in transformants only very rarely and this probably requires an additional mutational event to occur within the cells to allow immortalization [68,70]. It is not clear whether all the properties of Ad12E1B55K described in this set of experiments apply to E1B55K from other serotypes. There is detailed analysis of the relationship between Ad5E1B55K and components of the MRN complex, as well as other proteins involved in DSB repair, in Ad-infected cells but to what extent the protein itself affects the DDR is much less well understood. It might be supposed that the properties of Ad12 and Ad5E1B55K are broadly similar, since infection of human cells by both viruses results in degradation of many of the same DDR components, which is not the case for Ads from groups B, D, E and F [50,51]. However, even the Ad5 and Ad12 proteins share only about 50% homology, and so it is quite possible that they have unique features, including the ability of Ad12E1B55K to cause genetic instability. Indeed, it has been shown that the serotype of the E1B55K protein is the determining factor in E1-mediated tumor formation in athymic nude mice, such that rat cells transformed with Ad5E1A/Ad12E1B were as oncogenic as cells transformed with Ad12E1A/Ad12E1B, whereas cells with Ad5E1B55K were appreciably less oncogenic [71,72]. Furthermore, only group A adenoviruses cause tumors in new-born rodents. It is very likely that the genome-destabilizing properties of Ad12E1B55K described here contribute to this process.

The E1B55K interactome extends much more widely than the DDR (reviewed in [27]), and so it is likely that proteins known to associate but not investigated here contribute to some of the observed effects. However, it is probable that the genomic instability originally observed in human cells exposed to Ad12E1B55K could be explained by its effects on DNA replication and its interference with homologous recombination and possibly other DNA repair pathways.

## Data Availability

Data available in the published manuscript and in Supporting Material.

## Supplementary Materials

The following are available on line at https://www.mdpi.com/article/10.3390/v13122444/s1. Supplementary Figure S1. The expression od Ad12E1B55K in HSFs affects DNA replication. Supplementary Figure S2. R-loop detection. Supplementary Figure S3. Co-localization of Ad12E1B55K with repair foci after DNA damage. Supplementary Figure S4. Selected protein expression in HSFs and 55K^+^HSFs.
