# Supplementary material for: The Promotion of Genomic Instability in Human Fibroblasts by Adenovirus 12 Early Region 1B 55K Protein in the Absence of Viral Infection
Source: Viruses. 2021 Dec 6;13(12):2444. doi: 10.3390/v13122444 (PMC8708088; doi:10.3390/v13122444)
Supplement: Supplementary file 1 [file viruses-13-02444-s001.zip › viruses-1499715-supplementary.pdf]

Supplementary Figures

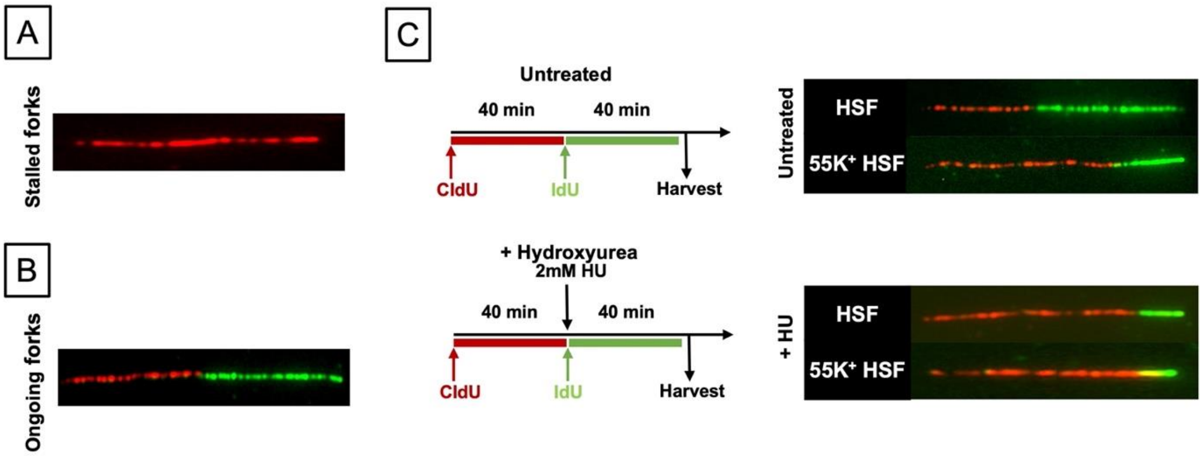

Supplementary Figure S1. Expression of Ad12E1B55K in HSFs affects DNA replication. DNA fibre assays. A, Examples of a stalled fork and B, an ongoing fork. C, summary of the labelling protocol with CldU and IdU in the presence and absence of HU. Examples of fibres observed in HSFs and 55K+HSFs in the presence and absence of hydroxyurea are shown.

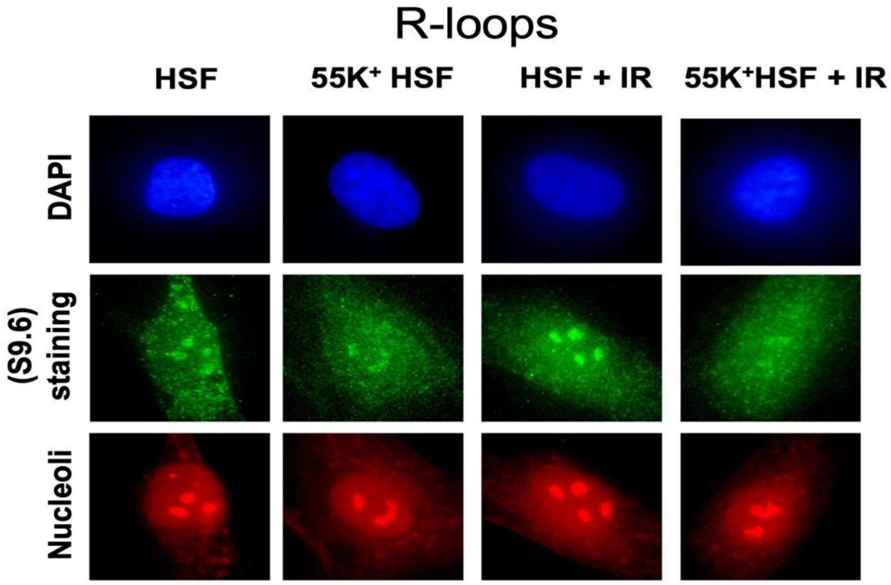

Supplementary Figure S2. R-loops detection. R-loop staining. Cells were stained with antibodies against R-loops (S9.6) and Nucleolin. Nuclei were stained with DAPI. The intensity of total nuclear staining due to S9.6 was measured; the intensity of S9.6 staining in the nucleoli was then subtracted as described [45].

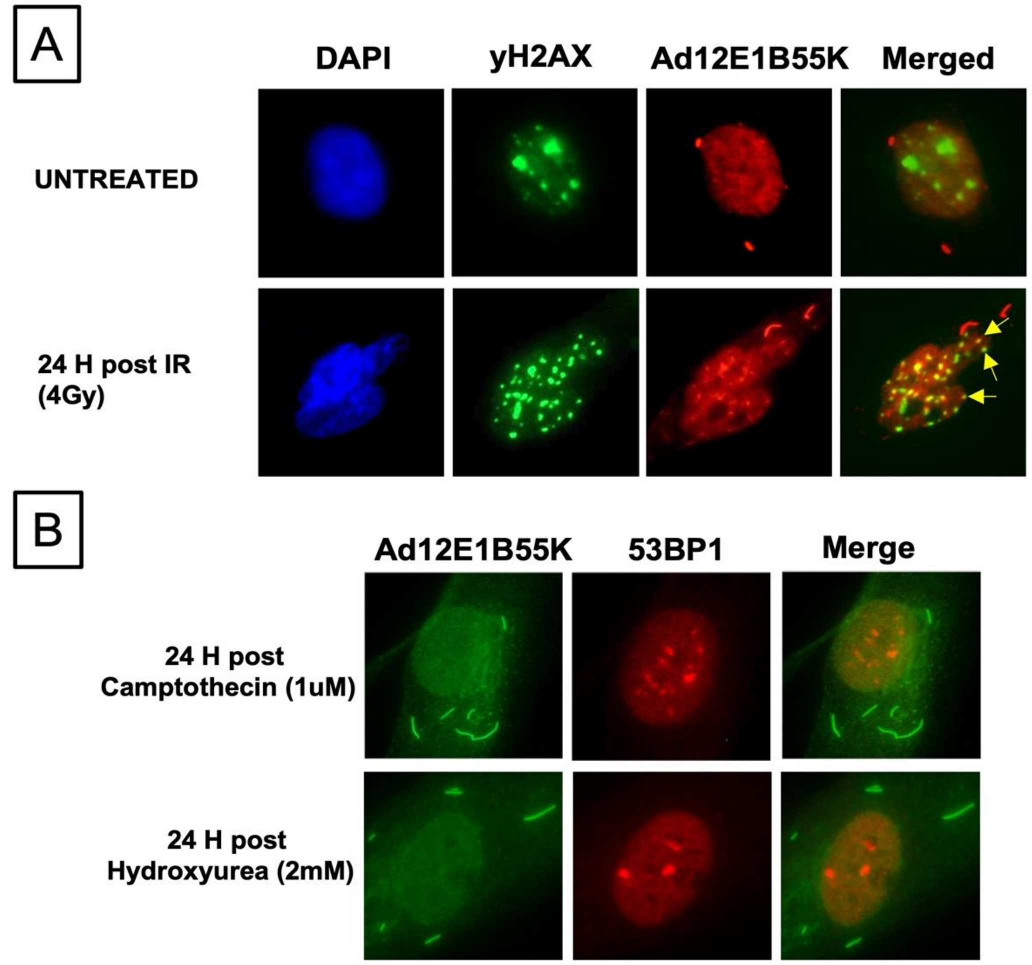

Supplementary Figure S3. Co-localisation of Ad12E1B55K with repair foci after DNA damage. Co-localisation of Ad12E1B55K with repair foci after DNA damage. 55K+HSFs were exposed to A, IR (4Gy) or B, camptothecin (1µM) or HU (2mM). Cells were fixed after 24 hours and then stained for Ad12E1B55K and counterstained for A, γH2AX or B, 53BP1. Rare possible examples of co-localization of Ad12E1B55K and γH2AX are indicated with arrows. No co-localization of Ad12E1B55K with 53BP1 bodies was observed.

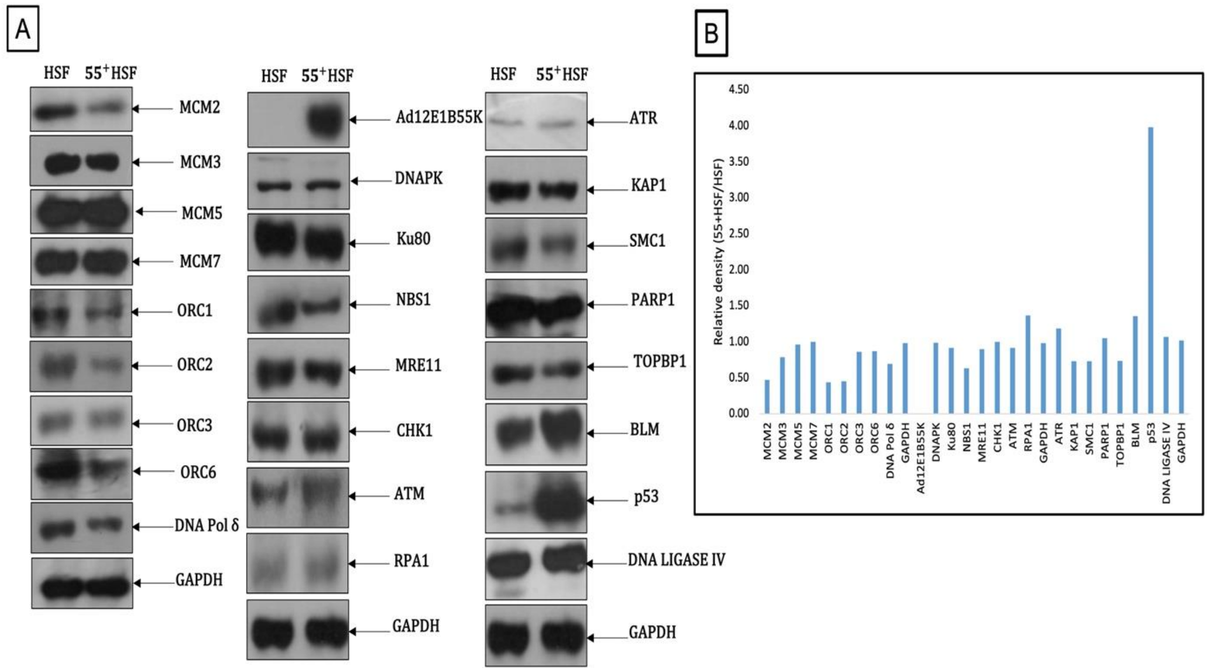

Supplementary Figure S4. Selected protein expression in HSFs and 55K+HSFs. Comparison of selected protein expression in HSFs and 55K$^+$HSFs. Cells were harvested, solubilized in 9M urea buffer and lysates fractionated by SDS.PAGE. A, western blotting was carried out using the antibodies shown. B, intensity of the bands was determined using ImageJ and the ratio of 55K+HSF to HSFs plotted.
